# Safety and efficacy of manual vacuum suction compared with conventional dilatation and sharp curettage and electric vacuum aspiration in surgical treatment of miscarriage: a randomized controlled trial

**DOI:** 10.1186/s12884-020-03362-4

**Published:** 2020-11-16

**Authors:** Toshiyuki Kakinuma, Kaoru Kakinuma, Yuuka Sakamoto, Yoshimasa Kawarai, Koyomi Saito, Motomasa Ihara, Yoshio Matsuda, Ikuo Sato, Michitaka Ohwada, Kaoru Yanagida, Hirokazu Tanaka

**Affiliations:** 1 Department of Obstetrics and Gynecology, International University of Health and Welfare Hospital, 537- 3 Iguchi, 329-2763 Nasushiobara City, Tochigi Japan; 2 Center for Human Reproduction, International University of Health and Welfare Hospital, 537-3 Iguchi, 329-2763 Nasushiobara City, Tochigi Japan

**Keywords:** Manual vacuum aspiration, Electric vacuum aspiration, Dilation and curettage, Asherman’s syndrome

## Abstract

**Background:**

The World Health Organization does not recommend dilatation and sharp curettage (D&C) for the surgical treatment of miscarriage during the first trimester because this may cause Asherman’s syndrome due to endometrial damage; therefore, suction remains the primary treatment option. While manual vacuum aspiration (MVA) has been widely used since the 1990s outside Japan, the use of an MVA device (Women’s MVA system) was approved in Japan in October 2015. Here, we examined the efficacy of the MVA kit in women surgically treated for miscarriage.

**Methods:**

This retrospective cohort study was conducted between 2014 and 2018 at the International University of Health and Welfare Hospital in Japan. Women who underwent surgical treatment for miscarriage within 12 weeks of pregnancy were identified and enrolled in the study. A total of 404 women were included who underwent the following procedures: 121 D&C, 123 electric vacuum aspiration (EVA), and 160 MVA. For each participant, the duration of surgery, amount of bleeding, amount of anesthetic used, incomplete abortion requiring repeat procedures, and intraoperative/postoperative complications were evaluated.

**Results:**

The duration of surgery was 13.7 ± 7.2, 11.2 ± 4.2, and 6.9 ± 4.3 min in the D&C, EVA, and MVA groups, respectively (*p* = 1.00). The amount of anesthetic used was not significantly different among all groups. Bleeding of ≥ 100 mL was confirmed in three (2.4%), one (0.8%), and one (0.6%) patient(s) in the D&C, EVA, and MVA groups, respectively (*p* = 0.50). Incomplete abortion was identified in three (2.4%), two (1.6%), and one (0.6%) patient(s) in the D&C, EVA, and MVA groups, respectively (*p* = 0.61). However, severe intraoperative/postoperative complications were not observed in any group.

**Conclusions:**

Surgical treatment for miscarriage performed using the MVA kit has safety and efficacy similar to those of conventional methods, such as D&C and EVA.

## Background

The World Health Organization (WHO) and the International Federation of Gynecology and Obstetrics (FIGO) recommend the use of suction for abortion as well as treatment of miscarriage during the first trimester from the perspective of safety, efficacy, and lower risk of endometrial damage, such as Asherman’s syndrome. These guidelines do not recommend the use of dilatation and sharp curettage (D&C); therefore, suction has become the mainstream treatment in Western countries, and manual vacuum aspiration (MVA) has been widely used since the 1990s. However, D&C is still performed in Japan in > 50% of cases [1].

In October 2015, an MVA device, called the Women’s MVA system, was approved for use in Japan and is being used in routine clinical practice. We introduced the use of the MVA kit at our hospital in June 2016 and have performed surgical treatments for miscarriage during the first trimester using the MVA kit only.

In the present study, we examined the efficacy and safety of MVA kit for the surgical treatment of miscarriage occurring during the first trimester.

## Methods

This retrospective cohort study was conducted between January 2014 and December 2018 at the International University of Health and Welfare Hospital in Tochigi, Japan. The study was approved by the Ethics Committee of the International University of Health and Welfare Hospital (referral number: 13-B-323). We excluded patients with contraindications to suspected ectopic pregnancy (no intrauterine pregnancy in the setting of complex adnexal mass or unilateral pain or ectopic pregnancy seen on ultrasonography), suspected molar pregnancy, and suspected completed spontaneous abortion (no gestational sac and vaginal bleeding). Women who underwent surgical treatment for miscarriage within 12 weeks of pregnancy were identified and enrolled in the study. Participants were randomized to one of the three study groups. We performed D&C alone between January 2014 and March 2015, electric vacuum aspiration (EVA) alone between April 2015 and May 2016, and MVA alone from June 2016 for the surgical management of early miscarriage at our hospital. All patients provided written and oral informed consent for the procedure and study participation after proper explanation of the risks and benefits of the procedure. In each case of surgical treatment for miscarriage, the duration of surgery, amount of bleeding, amount of anesthetic used, incomplete abortion requiring repeat procedures, and intraoperative/postoperative complications were evaluated.

Study participants were divided into three groups according to the treatment: D&C group, patients treated with D&C alone; EVA group, patients treated with EVA alone; and MVA group, patients treated with the MVA kit alone.

Surgery was performed using the J.A.M.W. placenta forceps and J.A.M.W. uterine curettes (Atom Medical Corp., Tokyo, Japan) in the D&C group, Atom Vacuum Extractor VP-450 (Atom Medical Corp.) in the EVA group, and Women’s MVA system MVA kit (Women’s Health Japan) in the MVA group. For nulliparous cases, the cervical canal was dilated prior to surgery using a Lamicel osmotic dilator (Medtronic, Tokyo, Japan). For all groups, we used intravenous anesthesia (15 mg of pentazocine and 1–1.5 mg of 1% propofol/kg of body weight). Additional 10–20 mg of propofol was administered if patients complained of intraoperative pain. In the D&C and EVA groups, we confirmed the position of the uterus and uterine cavity using a uterine probe and sequentially dilated the cervical canal using a J.A.M.W. cervical dilator (Atom Medical Corp.). In the D&C group, we used J.A.M.W. placenta forceps and J.A.M.W. uterine curettes according to the gestational age (size of the gestational sac). We extracted the uterine contents using J.A.M.W. placenta forceps and performed curettage with J.A.M.W. uterine curettes.

In the EVA group, we selected a metal catheter depending on the gestational age and suctioned the uterine contents with a pressure of − 400 to − 600 mmHg. After examination using a probe in the MVA group, the cervical canal was dilated to the diameter of the cannula equivalent to the gestational age (size of the gestational sac) using a dilator (diameter of 6 mm for 5–7 weeks, 7 mm for 7–8 weeks, 8 mm for 8–9 weeks, and 9 mm for 9–12 weeks), and the cannula was inserted into the fundus of the uterus. Subsequently, the double valve of the aspirator was closed, the plunger was removed to create a vacuum within the syringe (approximately pressure: −610 mmHg), and the uterine contents were suctioned after connecting with the inserted cannula. Surgery was performed in all patients under the guidance of transabdominal ultrasonography. Methylergometrine maleate was injected intramuscularly after confirming the complete removal of the uterine contents to complete the procedure, and all patients were administered oral methylergometrine maleate for 5 days postoperatively.

Student’s *t*-test and chi-squared test were performed for statistical analysis, and *P*- values of < 0.05 were considered statistically significant.

## Results

Study participants included 404 women who underwent surgical treatment for miscarriage within 12 weeks of pregnancy at our hospital between January 2014 and December 2018. Among these, 121 patients were included in the D&C group, 123 in the EVA group, and 160 in the MVA group.

**Fig. 1:**
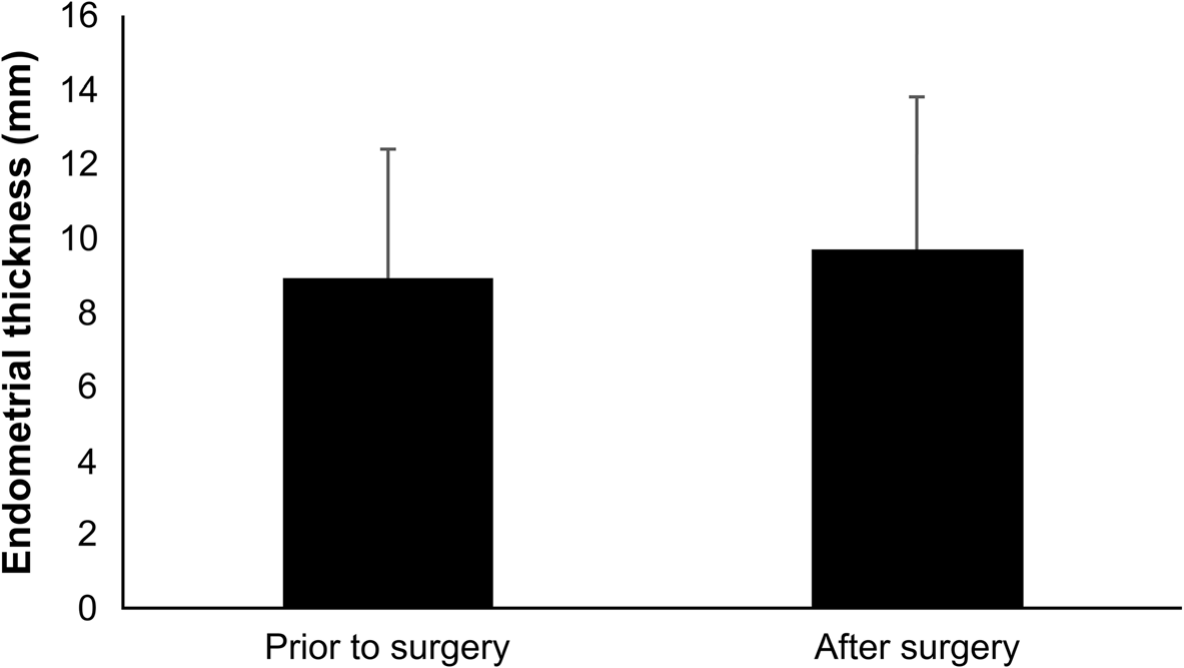
Comparison of endometrial thickness before and after MVA (represented in mean +/- SD). Endometrial thickness before and after miscarriage surgery from ovulation to implantation was compared in the MVA group and showed no thinning of the endometrium

Patient characteristics are presented in Table 1. The mean age of the study participants was 33.8 ± 5.4, 34.0 ± 5.1, and 35.3 ± 6.8 years in the D&C, EVA, and MVA groups, respectively (*p* = 0.44). The mean gestational age was 8.3 ± 1.3, 8.5 ± 1.4, and 8.4 ± 1.4 weeks in the D&C, EVA, and MVA groups, respectively (*p* = 0.84). Patient backgrounds were not significantly different among the groups (Table 1). The duration of surgery was 13.7 ± 7.2, 11.2 ± 4.2, and 6.9 ± 4.3 min in the D&C, EVA, and MVA groups, respectively (*p* = 1.00). The amount of propofol used was 9.6 ± 4.7, 9.0 ± 3.4, and 8.8 ± 2.5 mL in the D&C, EVA, and MVA groups, respectively (*p* = 0.76). Further, bleeding of ≥ 100 mL occurred in three (2.4%), one (0.8%), and one (0.6%) patient(s) in the D&C, EVA, and MVA groups, respectively (*p* = 0.50). Incomplete abortion was identified in three (2.4%), two (1.6%), and one (0.6%) patient(s) in the D&C, EVA, and MVA groups, respectively (*p* = 0.61). Surgical performance was not significantly different among the three groups (Table 2). Remarkably, no severe complications, such as damage to the uterus or cervical canal and uterine perforation were observed (Fig. 1)

**Table 1:** Patient background in the D&C, EVA, and MVA groups

	**D&C group (*n*= 121)**	**EVA group (*n*= 123)**	**MVA group (*n*= 160)**	***P*-value**
Age (years)	33.8 ± 5.4	34.0 ± 5.1	35.3 ± 6.8	0.44
Pregnancy history (cases)				0.1
Primigravida	60	63	98	
Multigravida	61	60	62	
Gestational age (weeks)	8.3 ± 1.3	8.5 ± 1.4	8.4 ± 1.4	0.84

Values expressed as mean ± standard deviation

*D&C* Dilatation and sharp curettage, *EVA* Electric vacuum aspiration, *MVA* Manual vacuum aspiration

**Table 2:** Surgical performance in the D&C, EVA, and MVA groups

	**D&C group (*n*= 121)**	**EVA group (*n*= 123)**	**MVA group (*n*= 160)**	***P*-value**
Duration of surgery (min)	13.7 ± 7.2	11.2 ± 4.2	6.9 ± 4.3	1.00
Amount of propofol used (mL)	9.6 ± 4.7	9.0 ± 3.4	8.8 ± 2.5	0.76
Bleeding of ≥100 mL (number of patients)	3 (2.4%)	1 (0.8%)	1 (0.6%)	0.50
Incomplete abortion (number of patients)	3 (2.4%)	2 (1.6%)	1 (0.6%)	0.61

Values expressed as mean ± standard deviation

*D&C* Dilatation and sharp curettage, *EVA* Electric vacuum aspiration, *MVA* Manual vacuum aspiration

## Discussion

The development of MVA started as the utilization of a small flexible cannula with a syringe by Harvey Karman in 1961 [2]. It was initially used as an apparatus to adjust the menstrual cycle by endometrial detachment. Subsequently, with support from the United States Agency for International Development, the Ipas MVA was developed in 1973, and was widely used in the 1990s across 100 countries worldwide. In Japan, the Women’s MVA system was approved in October 2015 for use in surgical treatment for miscarriage during the first trimester. We performed surgical treatment for miscarriage during the first trimester using the MVA kit only since June 2016 and examined the efficacy and safety of this kit for the surgical treatment of miscarriage occurring during the first trimester. In the present study, we confirmed that the MVA kit-based surgery for early pregnancy has the same level of safety and efficacy as conventional abortion surgery, D&C, and EVA. Other reports comparing MVA kits with D&C and EVA have also been published.

Vacuum aspiration techniques include MVA and EVA. A large-scale study including 2,399 patients undergoing abortion at up to 6 weeks of gestation showed that the success rate of MVA was 99.2%, with only 6 patients requiring additional surgery (0.25%); thereby, indicating the efficacy of MVA [3]. Several comparative examinations of MVA and EVA in miscarriages during the first trimester have been performed. In a study including 1,726 cases of abortion at up to 10 weeks of gestation (1,002 cases of MVA and 724 cases of EVA), the proportion of cases requiring additional surgery as well as those experiencing complications, such as bleeding and uterine perforation, was 2.5% for MVA and 2.1% for EVA, indicating no significant differences between the two groups [4]. In these studies, as well as in the present study, MVA showed efficacy and safety similar to that of EVA.

Several studies have compared MVA and D&C. Verkuyl et al. conducted a randomized study including 357 patients with incomplete miscarriage prior to 18 weeks of gestation and showed that MVA was associated with significantly less bleeding and pain and a shorter duration of surgery than D&C. However, there was no significant difference in the incidences of uterine perforation and septicemia [5]. In a Japanese study on the safety of surgical abortion methods during the first trimester, the frequency of complications, such as retention of uterine contents, uterine perforation, and severe bleeding, was 0.6% for D&C and 0.1% for MVA. The frequency of complications associated with D&C was six times higher than that for MVA, indicating that MVA was safer than D&C [1].

Regarding the efficacy of MVA from the perspective of endometrial protection, Gilman et al. examined the occurrence of Asherman’s syndrome in 1,580 patients treated for miscarriage during the first trimester and confirmed Asherman’s syndrome in 6 out of 483 (1.2%) patients treated with D&C alone or with D&C and EVA. However, no cases of Asherman’s syndrome were reported in patients treated with MVA [6]. Another study, which classified adenomyosis diagnosed histologically by magnetic resonance imaging, examined the characteristics of each type and mechanisms of onset. Subtype 1 adenomyosis, wherein the endometrium continuously infiltrates the myometrium, showed a positive correlation with a history of endometrial curettage, and endometrial damage caused by curettage was shown to be the cause of adenomyosis [7]. A study examining surgical techniques for miscarriage in patients with adenomyosis based on the histological analysis of the extracted uterus showed more cases of adenomyosis in patients with miscarriage treated with D&C than in those treated with MVA [8]. Our previous examination of endometrial thickness before and after MVA confirmed that there was no endometrial thinning [9]. MVA has limited risk of damaging the endometrial basal layer because it does not require curettes, and the plastic cannula used for MVA has appropriate hardness and flexibility, which reduces the occurrence of Asherman’s syndrome caused by endometrial damage and thinning. Therefore, MVA as a method of surgical management of miscarriage is considered effective for endometrial protection.

Another study describing the advantages of MVA reported that it is silent (because it does not require electricity), can be performed as an outpatient procedure, and involves single-use equipment, which eliminates the need to clean and disinfect surgical tools; thereby, reducing the risk of infection [3].

In Japan, induced abortion during the first trimester is performed using D&C in 33% of cases and using D&C and EVA with a metal suction tube in 47% of the cases [1]. D&C is the most commonly performed procedure [1]; however, WHO recommends that vacuum aspiration should be performed for surgical treatment for miscarriage at 12–14 weeks of gestation, and that D&C should be replaced by vacuum aspiration [10]. D&C is rarely performed in Europe and America because of the possibility of developing Asherman’s syndrome [11]. MVA is considered an effective and safe method for miscarriage surgery in the early stages of pregnancy and is expected to gain popularity in Japan.

## Conclusions

Surgical treatment for miscarriage performed using the MVA kit is as safe and efficacious as conventional methods such as D&C and EVA.

## Data Availability

There are no other data other than the data provided in the paper.

## Abbreviations

ACOG: American College of Obstetricians and Gynecologists.
D&C: Dilatation and sharp curettage.
EVA: Electric vacuum aspiration.
FIGO: International Federation of Gynecology.
MVA: Manual vacuum aspiration.
WHO: World Health Organization.
